# Relationships between stenosis severity, functional limitation, pain, and quality of life in patients with cervical spondylotic radiculopathy

**DOI:** 10.55730/1300-0144.5842

**Published:** 2024-06-06

**Authors:** Aydın Sinan APAYDIN, Musa GÜNEŞ

**Affiliations:** 1 Department of Neurosurgery, Faculty of Medicine, Karabük University, Karabük, Turkiye; 2 Department of Physiotherapy and Rehabilitation, Faculty of Health Sciences, Karabük University, Karabük, Turkiye

**Keywords:** Cervical radiculopathy, spinal stenosis, functional limitation, cross-sectional area, pain, disability

## Abstract

**Background/aim:**

This study aimed to examine the relationships between severity of stenosis, pain, functional limitation, disability, and quality of life in patients with cervical spondylotic radiculopathy.

**Materials and methods:**

Patients (45 female, 19 male) with radiculopathy due to spondylotic changes in the cervical spine were included in this study. Stenosis severity (thecal sac cross-sectional area (CSA)), numbness, neck and arm pain severity, functional limitation (Cervical Radiculopathy Impact Scale), disability, and quality of life (EQ-5D-3L General Quality of Life Scale) were evaluated. The study was registered at ClinicalTrials.gov as NCT06001359.

**Results:**

According to CSA values, 28 (43.75%) patients had severe stenosis and 36 (56.25%) had moderate stenosis, and the average CSA was 81.65 ± 10.08 mm^2^. Positive correlations were found between both neck and arm pain and neck disability (r = 0.597, r = 0.359), and negative correlations were found for the General Quality of Life Scale index score and EQ-5D-3L visual analog scale (r = −0.787, r = −0.518). There were significant positive correlations between Cervical Radiculopathy Impact Scale subscales and severity of stenosis, neck and arm pain, numbness, and disability (p < 0.05 for all). A significant negative correlation was observed between Cervical Radiculopathy Impact Scale subscales and quality of life (p < 0.01). Stenosis severity was correlated with pain, neck disability, and quality of life (p < 0.01 for all).

**Conclusion:**

There are direct relationships between cervical spondylotic radiculopathy and neck and arm pain, numbness, disability, and quality of life. Additionally, an increase in the severity of cervical stenosis is associated with an increase in pain and disability.

## 1. Introduction

Cervical spinal stenosis (CSS) describes symptomatic cases involving an anatomic reduction in the size of the spinal canal. Degenerative changes in the spinal elements cause CSS. These changes, together with the neural foramina, narrow the cervical spinal canal and result in cervical spondylotic radiculopathy (CSR) [1–3]. Therefore, pain is the first symptom. If the discomfort extends to the arm and shoulder and is accompanied by any sensory problems or motor impairment, cervical radiculopathy should be considered [3,4].

Cervical stenosis due to spondylotic changes causes spinal cord compression, which causes radicular pain in turn [3]. However, patients with a narrowed canal without showing any symptoms have also been reported [5]. Therefore, the relationship between stenosis and radicular symptoms is unclear. Various measurements of dural sac diameters or cross-sectional areas (CSAs) have generally been used to assess stenosis severity [5,6]. However, the relationship between imaging results and the patient’s symptoms is unclear. It is noted that many patients with minimal findings on imaging have significant clinical results and vice versa. Therefore, a correlation of appropriate clinical and radiological findings is necessary for better interpretation of treatment [5,7]. Additionally, studies generally include imaging studies in lumbar spinal stenosis (LSS) [5,6]. Stenotic radiculopathy arising from CSS also causes symptoms in patients [3,6]. However, the lack of a relationship between radiological imaging and clinical symptoms in CSS is noteworthy.

The pain experienced by patients with radiculopathy may spread to the neck and extremities, leading to decreased quality of life and functional limitation [8,9]. In radiculopathy, pain and limitations in upper extremity functions can lead to disability [10]. Neck and arm pain may be moderate to severe in patients with radicular symptoms due to disc herniation and this may increase the level of disability [9]. However, the relationships between decreased functionality and increased pain, disability, and quality of life with aging have not been clearly defined in patients with radiculopathy due to stenosis. It is also stated that pain and disability may be related to the severity of stenosis [7]. However, this relationship has not been examined for the cervical region. Examining patients’ disability levels and quality of life and determining their relationships with stenosis severity is essential for optimal treatment processes [9,11,12].

Pain radiating to the neck and arms caused by cervical radiculopathy may result in an increase in the severity of disability [10,11]. However, evaluations of the limitation caused by radiculopathy have generally been insufficient because these evaluations have considered the disability associated with neck pain and not the arm/extremity pain caused by radiculopathy [10]. The Cervical Radiculopathy Impact Scale (CRIS), which measures the symptoms and limitations of patients with cervical radiculopathy due to pain, tingling sensation, and loss of sensation in the neck and arms, provides more impartial data for assessments [10]. It has been stated that the CRIS reveals the functional limitations of individuals more clearly in patients with radicular symptoms caused by a cervical disc [11].

Studies in the literature have evaluated pain, quality of life, and disability in patients with radiculopathy due to neck pain [8,9]. However, there is a lack of sufficient research on the correlation between pain caused by CSR due to stenosis and its impacts on functional limitation, disability, and quality of life in these patients. Data on the relationships between these parameters and the severity of cervical stenosis are also limited. Furthermore, the absence of a comprehensive outcome scale for assessing functional limitation caused by radicular pain has contributed to deficiencies in evaluating such limitations. In this context, the newly developed CRIS evaluates functional limitation associated with radicular pain. The present study aims to investigate the relationships between stenosis severity, pain, functional limitation, disability, and quality of life in patients with CSR.

## 2. Materials and methods

### 2.1. Participants

This study was planned as observational cross-sectional research and was carried out between December 2023 and March 2024. Patients (n = 66) who presented to Karabük University Training and Research Hospital’s Neurosurgery Clinic and were diagnosed with radiculopathy due to spondylotic changes in the cervical spine were included in the study. Inclusion criteria for the patients were as follows: age of >45 years, stenosis in the cervical region confirmed with magnetic resonance imaging (MRI), and presence of root symptoms such as pain, numbness, and weakness in the extremities consistent with MRI and computed tomography results. Patients with severe neurological deficits, spinal malignancies, or a history of cervical surgery in the last 12 months were excluded.

All procedures were in accordance with the ethical standards of the responsible committee on human experimentation (institutional and national) and with the 1975 Declaration of Helsinki, as revised in 2008. The study was approved by the university’s ethics committee (2023/1605) and informed consent was obtained from all participants. The study was registered at ClinicalTrials.gov as NCT06001359.

### 2.2. Study design

After the neurosurgeon’s examination, the patients were asked to answer the questionnaire one-on-one. Demographic information was recorded. Three different numerical rating scale (NRS) scores were used for pain assessment: NRS Neck, NRS Arm, and NRS Numbness. The CRIS was used to evaluate radiculopathy, the Neck Disability Index (NDI) was utilized to evaluate the effects of neck pain on activities of daily living, and the EuroQol Five-Dimensions–3-Level (EQ-5D-3L) General Quality of Life Scale was applied to assess quality of life. Subsequently, MRI images were examined and the severity of stenosis was determined.

### 2.3. Outcome measures

#### 2.3.1. Magnetic resonance imaging measurements

All patients underwent a noncontrast cervical spinal MRI scan. The MRI scans included sagittal and axial T1- and T2-weighted images between the first cervical and first thoracic spinal segments. The anteroposterior (AP) diameter of the spinal canal and thecal sac CSA were measured using sagittal and axial sections of all cervical intervertebral levels. The smallest diameter measured among all levels was recorded. An AP diameter of the cervical canal of less than 10 mm was considered absolute stenosis, 10–13 mm was considered relative stenosis, and more than 13 mm was considered normal [6]. Thecal sac CSA of less than 75 mm^2^ was considered severe stenosis, 75–100 mm^2^ was considered moderate stenosis, and more than 100 mm^2^ was considered normal [5,6].

#### 2.3.2. Pain severity and numbness

NRS scores were used to measure pain intensity. They ranged from 0 (no pain/numbness) to 10 (worst pain/numbness imaginable). Patients were asked to mark their pain on a scale between 0 and 10 points. Neck pain, pain radiating to the arm, and pain and numbness in the fingers, hand, or arm were evaluated [12].

#### 2.3.3. Functional limitation

The Turkish version of the CRIS was used to determine functional limitation. This scale includes 21 questions within three categories. The first is symptoms, consisting of a total of nine items and assessing pain in the neck, shoulders, and arms/hands/fingers, as well as neck tingling, loss of strength, and stiffness. Second, energy and stances are assessed with 6 items, and the third category includes 6 items that evaluate how pain and discomfort have limited one’s ability to perform certain behaviors and activities [10,12].

#### 2.3.4. Neck disability

The NDI was used to evaluate the impact of disability caused by neck pain on functions. This scale consists of 10 items, including the severity of pain, headache, lifting, reading, concentration, personal care, work life, driving, sleep, and leisure activities. There are 6 possible answers for each item, ranging from 0 points to 5 points. As scores increase, disability increases. The Turkish validity and reliability of the scale were evaluated by Aslan et al. [13].

#### 2.3.5. Quality of life

The EQ-5D-3L scale was used to investigate quality of life. The first main section of this scale is the EQ-5D index scale. It comprises five dimensions: movement, self-care, usual activities, pain/discomfort, and anxiety/depression. These are scored with 1 (“no problem”) to 3 (“extreme problem”) points. An index score between −0.59 and 1 is calculated from the five dimensions of the scale. An index score of 1 indicates perfect health, while 0 points indicate death and negative scores reflect a state “worse than death.” Index scores were calculated according to Dolan et al. [14]. The second main section of the scale is the EQ-5D-3L visual analog scale (VAS). It entails a VAS that includes values between 0 and 100, and individuals evaluate themselves according to their perceived health on the day of the evaluation [14].

### 2.4. Statistical analysis

Power analysis was performed using the G*Power program. For a moderate correlation between pain and disability (r = 0.417), it was calculated that at least 57 people should participate in the study for a 95% confidence interval and 95% power, taking a previous study as a reference in determining the sample size [15]. Considering the possibility of data loss in the study, a minimum of 63 people were asked to participate for the 10% cutoff point.

The compliance of the data to normal distribution was evaluated with the Shapiro–Wilk test and histogram graphics. Descriptive analyses were shown with number and percentage values for categorical variables, mean and standard deviation, and median, minimum, and maximum values for numerical variables. Relationships between data were obtained with the Spearman correlation test. Correlation coefficients of r > 0.7 were considered to reflect strong correlations, 0.3–0.7 moderate correlations, and 0–0.3 weak correlations [16]. The level of statistical significance was set at p < 0.05.

## 3. Results

Sixty-six patients with CSR were screened and two were excluded from the study. One of those patients had undergone cervical spine surgery 6 months previously and one declined to participate in the study. The study was completed with a total of 64 patients. Of those 64 patients, 45 (70.30%) were female, 19 (29.70%) were male, and the mean age was 65.98 ± 3.77 years (Table 1).

The median duration of symptoms due to cervical radiculopathy was 21.48 months. Physical and demographic information about the patients is provided in Table 1. According to the NRS results, the patients had moderate pain in the neck and arm (5.51 ± 1.82 and 4.97 ± 1.65, respectively), and the mean NRS score was 4.20 ± 0.97 for numbness in the arm, hand, or fingers (Table 2). According to AP diameters, 44 (68.75%) patients had absolute stenosis, 20 (31.25%) had relative stenosis, and the average AP diameter was 9.36 ± 1.90 mm. In addition, according to thecal sac CSA, 28 (43.75%) patients had severe stenosis, 36 (56.25%) had moderate stenosis, and the average CSA was 81.65 ± 10.08 mm^2^. Data from the evaluations of the patients regarding severity of stenosis, neck and arm pain, CRIS subscales, neck disability, and health-related quality of life are provided in Table 2.

Correlation analysis revealed significant positive correlations between CRIS subscales and neck and arm pain, numbness, and disability (p < 0.05 for all). Significant negative correlations were observed between the CRIS subscales and both thecal sac CSA and health-related quality of life (p < 0.01 for both). The associations between CRIS subscales and other parameters are shown in Table 3.

Moderate positive correlations were found between both neck and arm pain and neck disability (r = 0.597, r = 0.359), and negative correlations were found for the EQ-5D-3L index score and EQ-VAS (r = −0.787, r = −0.518). There were moderate negative relationships between stenosis severity and pain, disability, and quality of life (p < 0.05 for all). The relationships between pain, numbness, disability, and quality of life are presented in Table 4.

## 4. Discussion

This study has shown that patients with CSR had moderate pain and numbness in their arms and neck. Functional limitation due to radiculopathy is associated with pain, disability, and quality of life. Additionally, it was determined that increased stenosis severity was associated with increased pain, functional limitation, and decreased quality of life.

CSR is a disease that generally progresses insidiously. Cervical spondylotic degeneration can be asymptomatic, although imaging methods show it clearly. Patients may complain of neck pain and numbness in their fingers and toes [3,15]. Most patients can adapt to the first signs of the disease over time. However, as the severity increases, the symptoms may manifest more [19]. The age group most affected by the disease is ≥55 years. More than 50% of the middle-aged population has radiological findings of cervical deterioration [18].

The natural course of CSR usually manifests itself in one of three ways. Up to 70% of patients show a slow worsening over time. The worsening period is followed by a relatively stable period; over time, repeated and slowly worsening attacks occur. This situation may continue cyclically and progressively [19]. Clearer progressive worsening is seen in 20% of patients, while a small group of 5% of patients show rapid deterioration. In 80% of cases, patients may remain stable for 3 years after diagnosis. Although some patients remain clinically stable in long-term follow-up, the majority will worsen clinically if untreated. Since CSR results from a degenerative process that increases with aging, its severity increases over time [19–21]. This may be associated with clinical symptoms.

Relationships between radiological images and clinical symptoms regarding the severity of stenosis have been investigated in the literature. However, these studies have generally focused on LSS [5,6]. It has been stated that significant clinical consequences may be observed with minimal findings in radiological images and vice versa [5]. Compared to patients with severe central stenosis, individuals with moderate central stenosis experienced more leg pain and had shorter maximal walking distances [5]. In another study of patients with LSS, no significant correlation was found between radiologically demonstrated anatomical LSS and disability scores [6]. Therefore, the results in the literature are contradictory.

Furthermore, the relationships between CSS-related radiological imaging and clinical symptoms have not been adequately investigated. One study stated that arm and neck pain was related to the severity of stenosis in patients with foraminal stenosis in the cervical region. However, no relationship was shown between stenosis severity and NDI [22]. Similarly, in the present study, it was observed that there was a linear relationship between the severity of stenosis and arm and neck pain according to thecal sac CSA. In contrast to Lee et al. [22], this study found an association between stenosis severity and neck disability. The inclusion of individuals with central stenosis in this study may explain that result. Additionally, the severity of stenosis increases with age [19–21]. The older ages of the patients in this study may explain the relationships observed between severity and clinical symptoms. In addition, with flexion and extension movements in the neck, a narrowing of the spinal canal of up to 2 mm in diameter may occur under physiological conditions. In this case, the dynamic process may increase the pressure on the spinal canal [21]. More use and movement of the neck compared to the low back area in daily life will cause radicular symptoms to increase due to compression [3,17]. Therefore, unlike LSS, a relationship may arise between the severity of stenosis in the cervical region and the patient’s symptoms. Deterioration in cervical alignment in the cervical region, which is less stable than the lumbar region because of stenosis, may also cause this finding.

Functional scales are important in determining the prognosis of CSR [10]. There needs to be a clear consensus on which test is adequate and useful in predicting prognosis. In previous studies, the NDI, the Nurick scale, and the 30-m walking test were used to follow patients and determine prognosis. Additionally, studies have noted the importance of the quality-of-life index in comparing treatment strategies for patients with CSR and evaluating the pre- and postoperative periods. Quality-of-life data and NDI values are valid and sensitive in evaluating and grading CSR patients [23]. However, the results obtained from the CRIS may give clearer information about these patients. There is a lack of research evaluating the relationships between pain caused by cervical radiculopathy due to stenosis and the limitations it causes [24]. The lack of an outcome scale that allows a comprehensive evaluation of the functional limitation caused by radicular pain has resulted in a deficiency in evaluating that limitation [25]. The results obtained from the CRIS in our study showed that patients with stenotic radiculopathy had moderate limitation. In a study evaluating functional limitation due to discogenic radiculopathy, lower scores were obtained compared to our study [9]. In another study, in contrast to the present study, it was stated that patients’ CRIS scores were close to each other but lower. In addition, a moderate relationship between functional limitation and disability was established [11], as in the present study. Stenotic changes increase with age and affect the functions of patients more. In this study, the patients’ higher levels of functional limitation may have been due to older age and, therefore, more stenotic problems.

This study also found that patients with stenotic radiculopathy had moderate shoulder and arm pain and moderate numbness in the arms. Similarly, a previous study showed that patients with cervical foraminal stenosis experienced moderate neck and arm pain [22]. Cervical spondylosis is caused by aging and it is a degenerative disease that can also affect the cervical vertebrae, facet joints and surrounding joints, and their associated soft tissues [23,24]. These changes lead to an increase in pain with an increase in pressure on the nerves. This explains why patients have moderate pain.

Evaluation methods are important in the management of radiculopathy for determining effective treatment and identifying whether the problems are due to radiculopathy [26]. The literature reports that patients with radiculopathy experience limitations due to increased pain intensity and disability symptoms [10,12]. However, since the evaluations consider the disability associated with neck pain rather than arm and extremity pain caused by radiculopathy, they are insufficient for evaluating the limitation caused by radiculopathy [10]. In the present study, significant positive correlations were determined between CRIS subscales and neck and arm pain, numbness, and disability. Significant negative correlations were also observed between CRIS subscales and health-related quality of life. The relationship between cervical stenosis severity and functional limitation has not been demonstrated before. This study revealed that an increase in functional limitation was associated with an increase in the severity of cervical stenosis. Therefore, evaluating the severity of functional limitation and stenosis in patients during clinical follow-up is essential.

Quality of life reflects the health status perceived by patients. It is also helpful in assessing the impact that dysfunctions resulting from disease or injury have on activities [27]. This study found moderate positive correlations between neck and arm pain and neck disability, and negative correlations were found for the EQ-5D-3L index score and EQ-VAS. A previous study showed that patients with discogenic radiculopathy had better quality of life and level of disability related to upper extremity function. Accordingly, disability may result from neck pain and deficiencies in upper extremity functions [27] and this situation may affect quality of life [15,27]. Conversely, decreased stenosis severity is associated with increased quality of life. The decrease in pain and disability may explain this [27]. Considering these results, quality of life is related to the severity of stenosis, functional limitation, pain, and disability.

This study has several limitations. First, it is possible that the radiological assessment of stenosis was inadequate because the MRI scans were obtained with patients in the supine position. In stenotic patients, symptoms typically worsen in the upright position [3,17]. Furthermore, the study was conducted in a single center. Multicenter studies are needed to generalize the results. Another limitation is that this study was cross-sectional. Studies aimed at treatment according to the degree of CSS could provide better results in confirming symptomatic levels.

## 5. Conclusion

This study has shown direct correlations between CSR and pain, numbness, disability, and quality of life. There are also relationships between stenosis severity, functional limitation, pain, and quality of life. Therefore, evaluation of radiculopathy-related symptoms and their relationships with the severity of stenosis is necessary for early and effective treatment in older adult patients.

## Figures and Tables

**Table 1:** Demographic and physical characteristics of the patients.

Variables	Total samples (n=64)	
Age (years) (X±SD)	65.98±3.77	
Height (cm) (Median (min–max))	162.36 (150.00–182.00)	
Weight (kg) (X±SD)	77.73±13.75	
Body mass index (kg/m^2^) (X±SD)	29.54±5.18	
Disease duration (month) (Median (min–max))	21.48 (3.00–72.00)	
	n	%
Sex		
Female	45	70.30
Male	19	29.70
Smoking status		
Current smoker	20	31.30
Ex-smoker	13	20.30
Nonsmoker	31	48.40
Dominant hand (%)		
Right	55	85.90
Left	9	14.10
Comorbidities		
No diseases	32	50.00
Diabetes	3	4.68
Hypertension	19	29.68
Heart diseases	4	6.25
More than one	6	9.37
Affected side (n, %)		
Right	22	34.40
Left	26	40.60
Bilateral	16	25.00

**Table 2:** Severity of stenosis, pain, numbness, functional limitation, disability, and quality of life scores of the patients.

Variables	Mean ± SD	Median (min–max)
A-P diameter, mm	9.36±1.90	9.50 (4.65–12.98)
Thecal sac CSA, mm^2^	81.65±10.08	79.33 (64.48–97.85)
NRS-neck	5.51±1.82	5.75 (2.00–9.00)
NRS-arm	4.97±1.65	5.00 (2.00–8.00)
NRS-numbness	4.20±0.97	4.00 (2.00–7.00)
CRIS		
Symptoms subscale	59.85±18.60	59.72 (16.67–100.00)
Energy and postures subscale	61.97±24.32	66.66 (12.50–100.00)
Actions and activities subscale	26.56±26.24	18.75 (0.00–95.83)
NDI	19.78±8.34	18.00 (4.00–42.00)
EQ-5D-3L index score	0.60±0.21	0.68 (0.09–0.86)
EQ-VAS	54.76±16.50	50.00 (10.00–90.00)

A-P: anteroposterior, CSA: cross-sectional area, NRS: numeric rating scale, CRIS: Cervical Radiculopathy Impact Scale, EQ-5D-3L: EuroQol five-dimensions – 3-level, VAS: visual analog scale.

**Table 3:** The associations between the cervical radiculopathy impact subscales with severity of stenosis, numeric rating scale, disability, and quality of life.

Variables	Symptoms subscale		Energy and postures subscale		Actions and activities subscale	
	r	p	r	p	r	p
A-P diameter, mm	−0.305*	0.014	−0.302*	0.015	−0.258*	0.039
Thecal sac CSA, mm^2^	−0.262*	0.037	−0.333**	<0.01	−0.343**	<0.01
NRS-neck	0.442**	<0.01	0,542**	<0.01	0,503**	<0.01
NRS-arm	0.471**	<0.01	0.370**	<0.01	0.396**	<0.01
NRS-numbness	0.419*	0.41	0.391**	<0.01	0.323**	<0.01
NDI	0.618**	<0.01	0.524**	<0.01	0.460**	<0.01
EQ-5D-3L index score	−0.541**	<0.01	−0.496**	<0.01	−0.341**	<0.01
EQ-VAS	−0.502**	<0.01	−0.526**	<0.01	−0.488**	<0.01

A-P: Anteroposterior, CSA: cross-sectional area, NRS: numeric rating scale, CRIS: Cervical Radiculopathy Impact Scale, EQ-5D-3L: EuroQol Five-Dimensions – 3-level, VAS: visual analog scale, r: Spearman correlation coefficient (*p < 0.05; **p < 0.01).

**Table 4:** The associations between severity of stenosis, pain, numbness, disability, and quality of life.

	Variables		1	2	3	4	5	6	7
1	A-P diameter, mm	r	1						
		p							
2	Thecal sac CSA, mm^2^	r	0.647**	1					
		p	<0.01						
3	NRS-neck	r	−0.257*	−0.438**	1				
		p	0.040	<0.01					
4	NRS-arm	r	−0.181	−0.350**	0.643**	1			
		p	0.152	<0.01	<0.01				
5	NRS-numbness	r	−0.216	−0.243	0.482**	0.711**	1		
		p	0.086	0.053	<0.01	<0.01			
6	NDI	r	−0573**	−0.569**	0.597**	0.359**	0.318*	1	
		p	<0.01	<0.01	<0.01	<0.01	0.011		
7	EQ-5D-3L index score	r	0.409**	0.325**	−0.787**	−0.518**	−0.400**	−0.701**	1
		p	<0.01	<0.01	<0.01	<0.01	<0.01	<0.01	
8	EQ-VAS	r	0.487**	0.593**	−0.431**	−0.271*	−0.337**	−0.741**	0.541**
		p	<0.01	<0.01	<0.01	0.030	<0.01	<0.01	<0.01

A-P: Anteroposterior, CSA: Cross-sectional area, NRS: Numeric rating scale, CRIS: Cervical Radiculopathy Impact Scale, EQ-5D-3L: EuroQol Five-Dimensions – 3-level, VAS: visual analog scale, r: Spearman correlation coefficient (*p < 0.05; **p < 0.01).

## References

[1] Hesni S Baxter D Saifuddin A The imaging of cervical spondylotic myeloradiculopathy Skeletal Radiology 2023 52 12 2341 2365 10.1007/s00256-023-04329-0 37071191

[2] Seo J Lee JW Magnetic resonance imaging grading systems for central canal and neural foraminal stenoses of the lumbar and cervical spines with a focus on the Lee grading system Korean Journal of Radiology 2023 24 3 224 234 10.3348/kjr.2022.0351 36788771 PMC9971835

[3] Kang KC Lee HS Lee JH Cervical radiculopathy focus on characteristics and differential diagnosis Asian Spine Journal 2020 14 6 921 930 10.31616/asj.2020.0647 33373515 PMC7788378

[4] Luyao H Xiaoxiao Y Tianxiao F Yuandong L Wang Ping Management of cervical spondylotic radiculopathy: a systematic review Global Spine Journal 2022 12 8 1912 1924 10.1177/21925682221075290 35324370 PMC9609507

[5] Kuittinen P Sipola P Saari T Aalto TJ Sinikallio S Visually assessed severity of lumbar spinal canal stenosis is paradoxically associated with leg pain and objective walking ability BMC Musculoskeletal Disorders 2014 15 348 10.1186/1471-2474-15-348 25319184 PMC4203914

[6] Goni VG Hampannavar A Gopinathan NR Singh P Sudesh P Comparison of the oswestry disability index and magnetic resonance imaging findings in lumbar canal stenosis: an observational study Asian Spine Journal 2014 8 1 44 50 10.4184/asj.2014.8.1.44 24596604 PMC3939368

[7] Minetama M Kawakami M Teraguchi M Matsuo S Sumiya T Endplate defects, not the severity of spinal stenosis, contribute to low back pain in patients with lumbar spinal stenosis Spine Journal 2022 22 3 370 378 10.1016/j.spinee.2021.09.008 34600109

[8] Liu J Kong Q Feng P Zhang B Ma J Analysis of the curative effect of cervical spondylotic radiculopathy with osseous foraminal stenosis using ultrasonic osteotome in anterior cervical surgery BMC Musculoskeletal Disorders 2023 24 1 81 10.1186/s12891-022-06083-1 36721172 PMC9887743

[9] Singh S Sathe PK Sathe A Kumar DV Evaluation of functional disability in cervical radiculopathy patients Indian Journal of Health Sciences and Biomedical Research 2023 16 103 110 10.4103/kleuhsj.kleuhsj_163_22

[10] Gärtner FR Marinus J van den Hout WB Vleggeert-Lankamp C Stiggelbout AM The Cervical Radiculopathy Impact Scale: development and evaluation of a new functional outcome measure for cervical radicular syndrome Disability and Rehabilitation 2020 42 13 1894 1905 10.1080/09638288.2018.1534996 30686066

[11] Thoomes E de Graaf M Gallina A Falla D Stathi A Comparison between two patient-reported outcome measures for patients with cervical radiculopathy: a think-aloud study Musculoskeletal Science and Practice 2023 65 102764 10.1016/j.msksp.2023.102764 37094507

[12] Çelenlioğlu AE Şencan S Saçaklıdır R Can Öztürk E Gündüz OH Cervical Radiculopathy Impact Scale: translation, cross-cultural adaptation, reliability and validity of the Turkish version Archives of Rheumatology 2022 37 4 574 583 10.46497/ArchRheumatol.2022.9639 36879564 PMC9985382

[13] Aslan ET Karaduman A Yakut Y Aras B Simsek IE The cultural adaptation, reliability, and validity of neck disability index in patients with neck pain: a Turkish version study Spine (Phila Pa 1976) 2009 34 16 1732 1735 10.1097/BRS.0b013e31817144e1 19770615

[14] Dolan P Gudex C Kind P Williams A The time trade-off method: results from a general population study Health Economics 1996 5 2 141 154 10.1002/(SICI)1099-1050(199603)5:2<141::AID-HEC189>3.0.CO;2-N 8733106

[15] Daliri BOM Khorasani HM Olia NDB Azhari A Shakeri M Association of psychological factors with limb disability in patients with cervical radiculopathy: comparison with carpal tunnel syndrome BMC Musculoskeletal Disorders 2022 23 1 667 10.1186/s12891-022-05593-2 35831834 PMC9281137

[16] Ratner B The correlation coefficient: its values range between +1/−1, or do they? Journal of Targeting, Measurement and Analysis for Marketing 2009 17 139 142

[17] Efstathiou MA Stefanakis M Savva C Giakas G Effectiveness of neural mobilization in patients with spinal radiculopathy: a critical review Journal of Bodywork and Movement Therapies 2015 19 2 205 212 10.1016/j.jbmt.2014.08.006 25892373

[18] Liu J Kong Q Feng P Zhang B Hu Y Clinical effect of channel assisted cervical key hole technology combined with ultrasonic bone osteotome in the treatment of single segment cervical spondylotic radiculopathy Frontiers in Surgery 2022 9 1029028 10.3389/fsurg.2022.1029028 36325044 PMC9618798

[19] Shi M Wang C Wang H Ding X Feng J Posterior cervical full-endoscopic technique for the treatment of cervical spondylotic radiculopathy with foraminal bony stenosis: a retrospective study Frontiers in Surgery 2023 9 1035758 10.3389/fsurg.2022.1035758 36684297 PMC9845617

[20] Zhang P Jin Y Zhu B Zheng M Ying X Unilateral biportal endoscopic foraminotomy and diskectomy combined with piezosurgery for treating cervical spondylotic radiculopathy with neuropathic radicular pain Frontiers in Neurology 2023 14 1100641 10.3389/fneur.2023.1100641 37114218 PMC10126746

[21] Liu ZQ Hsieh CT Huang CT Hsu SK Fang JJ Combining laminoplasty with artificial disc replacement for the treatment of cervical spondylotic myelopathy with congenital cervical stenosis International Journal of Spine Surgery 2023 17 4 492 501 10.14444/8475 37253625 PMC10478697

[22] Lee HD Jeon CH Chung NS Yoon HS Chung HW Is the severity of cervical foraminal stenosis related to the severity and sidedness of symptoms? Healthcare 2021 9 12 1743 10.3390/healthcare9121743 34946469 PMC8701450

[23] Turner I Choi D NuNec™ cervical disc arthroplasty improves quality of life in cervical radiculopathy and myelopathy: a 2-yr follow-up Neurosurgery 2018 83 3 422 428 10.1093/neuros/nyx424 28973309

[24] Carette S Fehlings MG Cervical radiculopathy The New England Journal of Medicine 2005 353 4 392 399 10.1056/NEJMcp043887 16049211

[25] Rafiq S Zafar H Gillani SA Waqas MS Liaqat S Effects of neurodynamic mobilization on health-related quality of life and cervical deep flexors endurance in patients of cervical radiculopathy: a randomized trial Biomed Research International 2022 2022 9385459 10.1155/2022/9385459 36246968 PMC9556173

[26] Liang L Feng M Cui X Zhou S Yin X The effect of exercise on cervical radiculopathy: a systematic review and meta-analysis Medicine 2019 98 45 e17733 10.1097/MD.0000000000017733 31702624 PMC6855577

[27] Demirel A Quality of life, disability, functional status, and relationship with radiculopathy in patients with cervical disc herniation Journal of Occupational Therapy and Rehabilitation 2019 7 3 123 128 10.30720/ered.468859

